# Fully Coupled Three-Dimensional Simulation of Downward Flame Spread over Combustible Material

**DOI:** 10.3390/polym14194136

**Published:** 2022-10-02

**Authors:** A. Snegirev, E. Kuznetsov, O. Korobeinichev, A. Shmakov, A. Paletsky, V. Shvartsberg, S. Trubachev

**Affiliations:** 1 Department of Structural Engineering (Combustion, Fire and Fire Safety), Faculty of Engineering and Architecture, Ghent University, 9000 Ghent, Belgium; 2 Autonomous Non-Profit Organization “Fire and Explosion Safety”, 199155 St. Petersburg, Russia; 3 Voevodsky Institute of Chemical Kinetics and Combustion, 630058 Novosibirsk, Russia

**Keywords:** counterflow flame spread, flammability, pyrolysis, laminar diffusion flame, coupled simulations

## Abstract

Three-dimensional simulations of laminar flame propagating downwards the vertical surface of a rigid polyurethane slab heated by a radiative panel are presented and compared with the measurement data. The gas-phase model (ANSYS Fluent) allows for finite-rate volatile oxidation, soot formation and oxidation, emission, transfer, and absorption of thermal radiation. The solid-phase model Pyropolis considers heat transfer across the material layer and generation of combustible volatiles in thermal decomposition of the material. Kinetic model of material decomposition is derived to obey the microscale combustion calorimetry data for different heating rates. Transient behavior of propagating flame and pyrolysis zone, as well as spatial distributions of heat flux components, temperature, and mass burning rates over the specimen surface are examined. Variation of the thermal properties of the material during its thermal decomposition, as well as the specimen surface emissivity and reradiation are shown to be the important issues strongly affecting model predictions. Two distinct modes of counterflow flame spread, thermal and kinetic, are identified. In the thermal mode corresponding to fast chemistry in the gaseous flame, the flame propagation velocity is governed by the heating rate of the combustible material ahead of the flame front. Alternatively, in the kinetic mode, it is limited by the burning velocity of the volatile-air mixture forming ahead of the flame front. Simulation results are favorably compared with the measured propagation velocity.

## 1. Introduction

Driving mechanisms and dynamics of concurrent and opposed flame spread over solid combustibles are markedly different [1], which implies the requirement for a comprehensive model to be capable of replicating both scenarios. This work continues the recent study, Ref. [2], with the overall aim of developing a comprehensive 3D computational methodology for high-resolution predictions of flame spread over combustible surfaces. In Ref. [2], the upward flame propagation was considered after the composite specimen was ignited by the adjacent flame. Upward flame propagation occurs when a concurrent airflow develops due to buoyancy. A downward flame propagation scenario, in which the flame edge moves towards an opposed airflow, is remarkably distinct due to the different heat transfer pathway from gaseous flame to the solid fuel. As a result, a downwardly propagating flame moves at a much lower speed, and the self-sustained flame propagation may require the initial material temperature and/or the external heating rate to be above certain critical values [1,3].

Here, we consider a downward flame propagation scenario and validate the modeling approach against the experimental data obtained in this study for a burning polyurethane slab. The literature review indicates that previous experience of modeling flame spread was restricted by the assumption of a two-dimensional flame and flow in the gas phase (e.g., [4,5,6,7,8,9]), and examples of three-dimensional simulations are still rare [10]. Although a consideration of the three-dimensional problem setup is essential for subsequent practical applications, the feasibility of this computational approach has not been sufficiently explored.

A comprehensive approach also implies a detailed consideration of heat transfer in and thermal degradation of the combustible layer, with the kinetics of pyrolysis reactions and the variation of thermal properties taken into account. In this work, we apply a computational framework that has been developed by combining the in-house model of material thermal decomposition (Pyropolis, see Refs. [2,11]) and ANSYS Fluent software. The focus of this study is the development of a computational framework for fully coupled and sufficiently resolved 3D simulations of counter-flow flame propagation. This methodology aims to be applied to a wide range of combustible polymers. The selection of a particular polymer material (rigid polyurethane foam) was motivated by the availability of the experimental data to validate this model.

## 2. Experiment

### 2.1. Flame Spread

The rigid polyurethane porous samples considered in this study were manufactured by the free expansion method. The mixture used in sample production included isocyanate (150 g), polyol (100 g), triethylenediamine (1 g), dibutyl tin dilaurate (0.5 g), water (2 g), silicone oil (2 g), and triethanolamine (3 g). Two primary components, isocyanate (PAPI, Wanhua Chemical Group Co., Ltd., Yantai, China) and polyol (LY-4110, Jiangsu Luyuan New Materials Co., Ltd., Nantong, Jiangsu, China), interact in the presence of catalysts with intensive foam formation. The sample density is 0.076 g/cm^3^, and the isocyanate index (mole NCO per mole OH) is 1.05.

**Figure 1:**
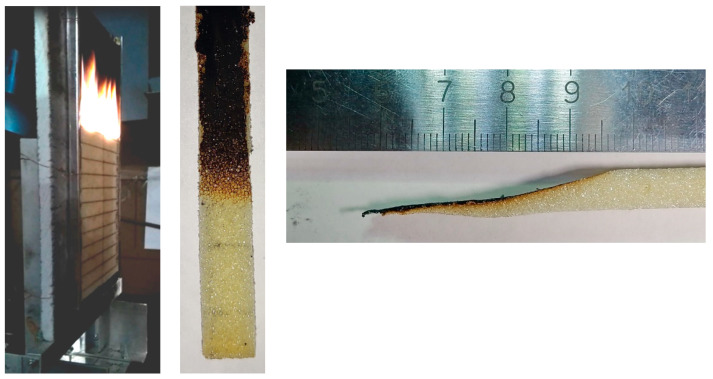
Experimental observation of the downward flame spread over the vertical polyurethane slab. **Left** to **right**: side view of the established propagating flame (horizontal lines are placed at a distance of 1 cm); front and side view of the extinguished specimen.

In the experiment, a 6.7 mm-thick, 19.8 cm-high, 9.9 cm-wide rigid polyurethane slab was vertically placed in an aluminum holder covering 5 mm vertical strips at the sides of the specimen. The rear side of the slab was attached to the plasterboard wall (see Figure 1, left). The slab was initially warmed up by the radiative panel until the rear and exposed surface temperatures approached their steady state values of about 62 and 82 °C. The panel was much larger in size than the specimen and was located 10 cm away from the exposed specimen surface. The specimen was then ignited by a pilot burner flame moved closely to the upper edge of the slab. After a few seconds of flame impact, the flame was removed, and the self-sustained flame propagated downwards over the specimen surface.

The flame propagated downwards until the exposed part of the specimen burned out completely, and the flame propagation velocity fluctuated around 1 mm/s. In a separate test, the specimen was extinguished when the flame front passed a halfway downwards. The front and side view of the extinguished specimen are shown in Figure 1 (middle and right).

### 2.2. Surface Incident Radiation and Surface Emissivity

**Figure 2:**
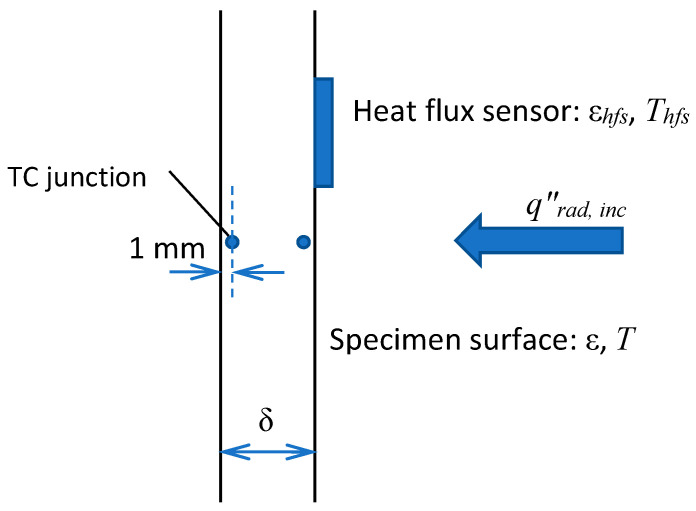
One-sided heating of the sample.

**Figure 3:**
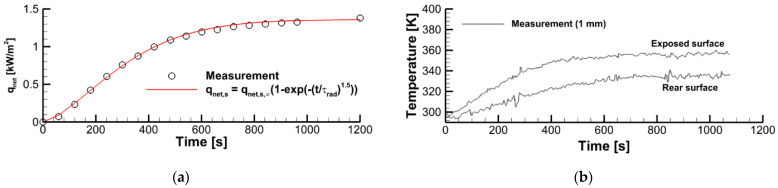
Transient variation of the net heat flux received by the sensor (**a**) and the thermocouple output (**b**).

To evaluate the surface incident radiation (coming from the radiative panel) and the sample surface emissivity, we used the measurement data obtained in a one-side sample heating with no ignition. As shown in Figure 2, the vertically positioned sample was exposed to the radiative flux, and the outputs of two thermocouples (type K, junctions are located at the distance of *δ_TC_* = 1 mm from the surface) and of the heat flux sensor (gSKIN^®^ Heat Flux Sensor) were recorded for 20 min after the radiative panel was activated. The measurement results are presented in Figure 3, wherein the net heat flux recorded by the sensor is shown in Figure 3a, and the thermocouple readings at the exposed and rear surfaces are provided in Figure 3b.

The heat flux recorded by the sensor includes the absorbed portion of incident radiation, surface re-radiation, and convective heat flux at the exposed surface of the sensor:

(1) $${q^{\prime \prime }}_{net,hfs}\left(t\right)=\varepsilon_{hfs}{q^{\prime \prime }}_{rad,inc}\left(t\right)-\varepsilon_{hfs}\sigma T_{hfs}^{4}-h\left(T_{hfs}-T_{0}\right)$$

According to Equation (1), the surface incident radiation is

(2) $${q^{\prime \prime }}_{rad,inc}\left(t\right)=\frac{1}{\varepsilon_{hfs}}\left({q^{\prime \prime }}_{net,hfs}\left(t\right)+\varepsilon_{hfs}\sigma T_{hfs}^{4}+h\left(T_{hfs}-T_{0}\right)\right)$$

where the subscript “*hfs*” indicates the exposed surface of the heat flux sensor. We assume that the surface incident radiation is the same at the exposed surfaces of both the sensor and the specimen. However, the net heat fluxes at these surfaces are different because of the different surface temperatures and emissivities. The net heat flux at the exposed specimen surface is:

(3) $${q^{\prime \prime }}_{net}\left(t\right)=\varepsilon {q^{\prime \prime }}_{rad,inc}\left(t\right)-\varepsilon \sigma T^{4}-h\left(T-T_{0}\right)$$

In the steady state (i.e., at t→∞), the net heat flux passing through the sample is ${q^{\prime \prime }}_{net,\infty }$ = $k\Delta T_{TC,\infty }/\left(\delta -\delta_{TC}\right)$, where $\Delta T_{TC,\infty }$ is the steady-state temperature drop between the thermocouple junction locations, and k is the thermal conductivity of the sample material. At the same time, the steady-state net heat flux is determined by the heat balance at the exposed surface:

(4) $${q^{\prime \prime }}_{net,\infty }=\varepsilon {q^{\prime \prime }}_{rad,inc}-\varepsilon \sigma T_{\infty }^{4}-h\left(T_{\infty }-T_{0}\right)$$

The measurement results presented in Figure 3 provide the following values in the steady state: ${q^{\prime \prime }}_{net,\infty }$ = 1360 B_T_/_M_^2^, $T_{0}$ = 295 K, $\Delta T_{TC,\infty }$ = 20 K, and $T_{\infty }$ = 350 K. Using $T_{hfs}$ = 303 K, $\varepsilon_{hfs}$ = 0.95 for the steady-state temperature and emissivity of the sensor surface, k = 0.026 W/(m·K) for the thermal conductivity of the sample material, δ = 0.0067 m for the sample thickness, and $\delta_{TC}$ = 0.001 m, we obtain for the steady-state heat flux recorded by the sensor ${q^{\prime \prime }}_{net,\infty }$ = $k\Delta T_{TC,\infty }/\left(\delta -\delta_{TC}\right)$ = 0.026·20/0.0047 = 111 W/m^2^.

The convective heat transfer coefficient, h, can be estimated by the empirical correlation for the free convection near a vertical slab, and it is expected to be about 10–20 W/(m^2^·K). Using these values and applying Equation (2), we obtain ${q^{\prime \prime }}_{rad,inc}$ = 1994 to 2078 W/m^2^ for the surface incident radiation at the exposed specimen surface. Using (3), we measure the specimen surface emissivity, $\varepsilon =\left({q^{\prime \prime }}_{net,\infty }+h\left(T_{\infty }-T_{0}\right)\right)/\left({q^{\prime \prime }}_{rad,inc}-\sigma T_{\infty }^{4}\right)$, to be in the range from 0.6 to 0.9.

These estimates indicate that the effective temperature of the radiative panel assumed in the simulations should be selected to provide the surface incident radiation at the specimen surface to be close to 2 kW/m^2^. The specimen surface emissivity is more uncertain, and it may also vary as the specimen ignites and burns. As such, the specimen surface emissivity is the effective quantity that can be used to calibrate predictions against the measurements. Since the flame is optically thin, its radiative impact on the sample surface is low, and the surface emissivity of the burning specimen mainly affects the heat loss due to surface re-radiation. Therefore, a higher value of the surface emissivity would cause a reduced burning rate and, therefore, a lower flame propagation velocity. This has been confirmed in the numerical simulations. The simulation results presented in this work were obtained with ε = 0.9.

## 3. Modeling and Simulations

### 3.1. Problem Setup

**Figure 4:**
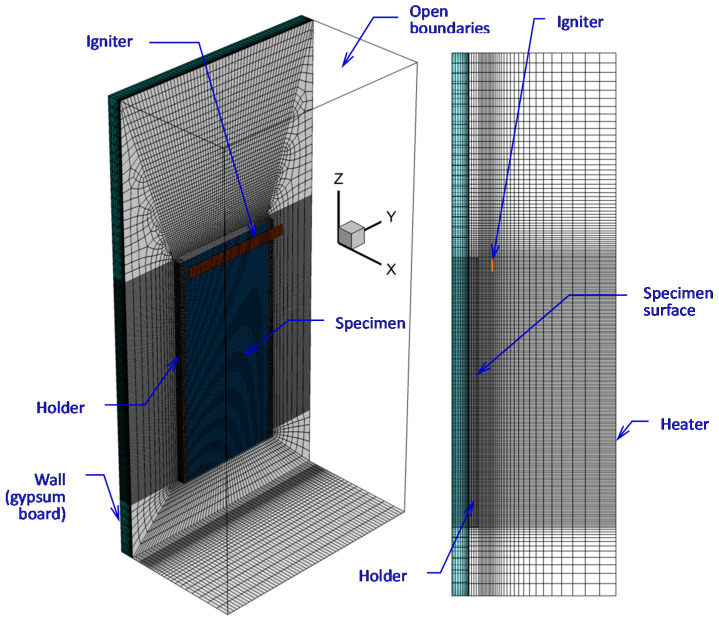
Computational domain and mesh.

In the simulations, we consider a vertical combustible slab (6.7 mm thick, 19.8 cm high, 8.9 cm wide) attached to a 5 mm aluminum holder at the sides and to a plasterboard wall at the rear surface (Figure 4). Conjugate heat transfer at the specimen-holder and specimen-wall interfaces is accounted for, and the temperature distributions in the holder and wall regions are evaluated. The entire side surface of the computational domain parallel to the exposed specimen surface and located at 10 cm away from the specimen is considered to be the surface of the radiant heater. The side and top boundaries of the computational domain are open to the incoming airflow and outgoing gas. The bottom boundary is the solid wall (6 cm below the lower specimen edge).

**Figure 5:**
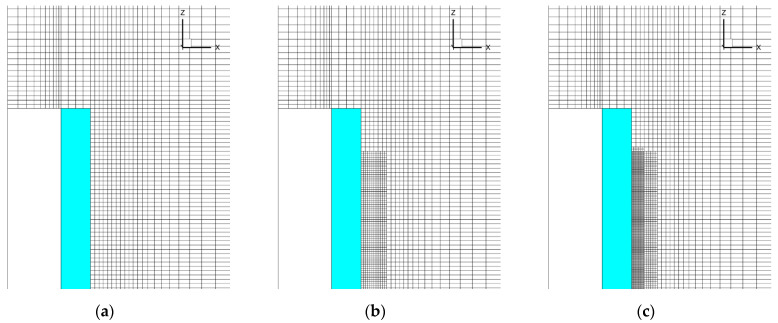
Three meshes used in the simulations: (**a**) basic mesh; (**b**) mesh 2; (**c**) mesh 3.

To evaluate the effect of spatial resolution in the gas phase, three meshes were applied in the simulations. The basic mesh was generated based on the requirement to resolve the temperature gradients in the flame (possibly, except for its leading edge) and at the specimen surface covered by the flame (see the details in Ref. [2]). In this mesh, the characteristic cell size near the specimen surface is 0.5 mm, and it was found to be sufficient to approximately replicate sample burning rates in *upward* flame spread [2]. This mesh is shown in Figure 4 and Figure 5a.

It appears, however, that a much stricter resolution is required to simulate the *downward* flame spread, which is directed opposite to the uprising buoyant flow. The first reason for this is that, in such a counterflow flame spread, the velocity of flame propagation is strongly affected (and might be fully controlled) by the heat flux from the flame edge to the preheat zone ahead of the flame. In its turn, this heat flux is mainly conductive and, therefore, requires an accurate resolution of the temperature gradient near the leading edge of the flame. Another important reason, which is often underestimated, is the need to also resolve the inner structure of the flame edge in the gas phase, including the reaction zone, which is thinner than the preheat zone. This becomes particularly important when the flammable mixture of volatiles with air forms ahead of the flame edge. In this case, the velocity of flame propagation over the solid combustible surface also depends on the burning velocity of this flammable mixture.

In view of the above arguments, the mesh refinement was undertaken in the region where the gaseous flame edge propagates over the surface. The meshes are demonstrated in Figure 5, which shows the vertical cross-section of the computational domain including the gas, the burning specimen, and the plasterboard substrate behind the specimen (the mesh is only shown in the gas region). The first mesh (Figure 5a) is the basic one described above. In the second mesh (Figure 5b), the near-sample region (up to 1 cm away from the exposed sample surface) is refined by splitting each cell of the basic mesh by two equal cells in each direction, thereby reducing the cell volume in this region by a factor of eight. In the third mesh, further halving of the cells (in three directions) is undertaken in the region near the sample surface up to 0.6 cm away from the surface (Figure 5c). The total amount of the cells in these meshes was 750732, 1177116, and 2893944, respectively.

We found that, with the input data used in this work, a steady flame propagation over the entire sample is not predicted with the basic mesh. With this mesh, the simulated flame does not propagate beyond the region of about 2 cm that starts burning when the igniter is on. After the igniter is turned off, the flame is only predicted to exist until the sample burns out in this region, ceasing shortly after that without spreading downwards. The dynamics of the flame propagation predicted with two refined meshes is demonstrated in Figure 5 by transient dependences of flame front coordinates at the centerline of the burning surface.

**Figure 6:**
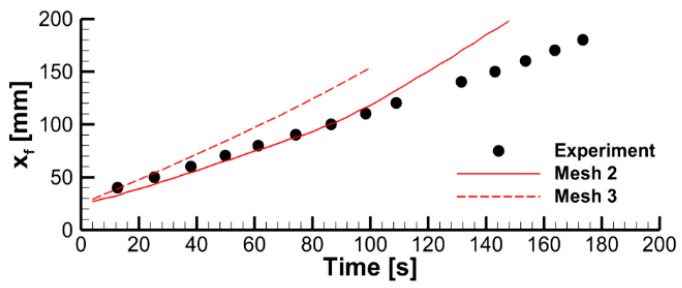
The effect of mesh refinement on predicted flame propagation dynamics. Flame front coordinate is defined as the distance from the upper edge of the specimen to the flame front at the vertical centerline.

**Figure 7:**
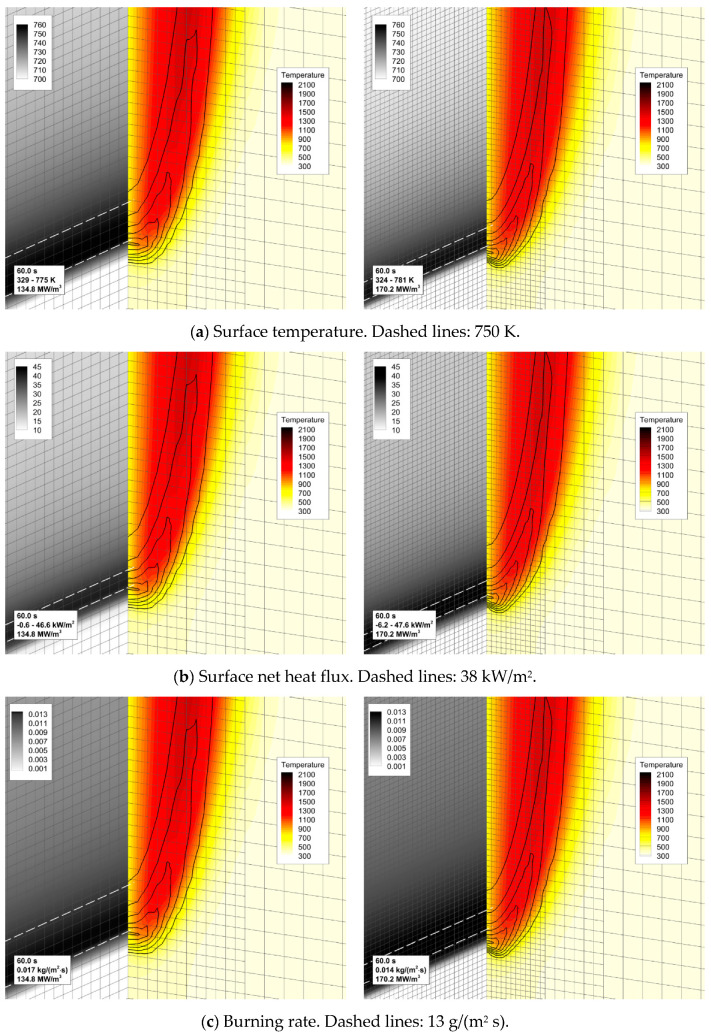
The effect of mesh refinement on flame structure and sample surface distributions near the edge of propagating flame. Distributions of (**a**) temperature, (**b**) net heat flux, and (**c**) volatile emission rate per unit area (burning rate) over the sample surface are shown along with the distributions of gas temperature (color) and volumetric heat release rate (lines) in the vertical plane containing the sample centerline. **Left**—mesh 2, **right**—mesh 3.

The predictions are not perfectly mesh-independent, and a certain increase of the predicted flame propagation velocity is still expected in case of further mesh refinement. However, in view of the drastically increased computational time, it was decided to proceed with the simulations made with the intermediate mesh (mesh 2 in Figure 5b). This decision is supported by the observation that, with this mesh, the experimentally observed flame propagation dynamics is reproduced reasonably well (see Figure 6). It was also found that the flow structure in the gas phase predicted with meshes 2 and 3 remains similar, as well as the distributions of temperature, heat fluxes, and volatile emission rate over the sample surface (Figure 7).

It is worthy of note that flame propagation velocities predicted with different meshes differ despite the corresponding net heat fluxes being practically indistinguishable. This is an indication of the flame spread phenomenon being controlled not only by the rate of the sample heating but also by the spatial resolution of the gaseous flame (both the temperature gradients and the reaction zone). Resolution of the reaction zone affects the accuracy at which the velocity of the gaseous flame propagation (gas burning velocity) is predicted. The need to resolve the reaction zone of the gaseous flame makes the task of simulating the counterflow flame spread more demanding than that in the case of the co-flow flame spread.

**Figure 8:**
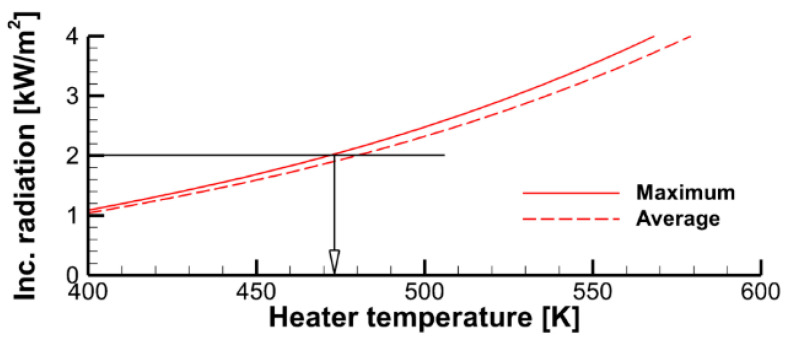
Maximum and surface-averaged incident radiative flux at the specimen exposed surface as a function of the heater temperature.

The computational domain shown in Figure 4 does not replicate the geometry of the heater (radiative panel) used in the experiment. Furthermore, the problem setup assumes a uniform temperature distribution over the radiating surface, thereby ignoring the non-uniformity of the temperature distribution over the real panel. Therefore, to provide the surface incident radiation in accordance with the above estimates (about 2 kW/m^2^, as derived from the requirement to maintain the steady-state temperature of the exposed specimen surface close to 350 K), the effective surface temperature of the heater should be selected. The calculated dependence of the surface incident radiation at the exposed specimen surface on the heater temperatures is demonstrated in Figure 8, which shows that heater temperatures of 450–470 K cause this requirement to obey for either the maximum or surface-averaged value of the incident radiative flux at the sample surface.

In the simulations (with the exception of those presented in Section 4.3), we assume the initial specimen temperature to be uniform across the specimen and set it equal to 350 K, which is the average of the steady-state surface temperatures recorded in the specimen warming prior to ignition. The temperature of the heater was set equal to 470 K (uniform over the radiating surface).

Ignition (by pilot burner flame in the experiment) was modeled by the radiative flux produced by the hot black strip (9.9 cm wide, 1 cm high) located at a distance of 1 cm away from the exposed specimen surface, with the upper edge of the igniter being at the same elevation as the upper edge of the specimen (see Figure 4). The igniter temperature was set 1200 K for 4 s, and this boundary condition was switched to the condition of zero heat flux afterwards. A zero-stress boundary condition was set for velocity at the igniter surface. The simulations have shown that the presence of the igniter did not significantly affect the flow after ignition, particularly after the flame front moved away.

An alternative, albeit similar, problem setup included preliminary simulation of the specimen heating until the steady state was established without the ignitor, followed by switching the igniter on for 4 s as highlighted above (see Section 4.3).

### 3.2. Solid Phase Model

#### 3.2.1. Heat and Mass Transfer

Heat transfer across the material layer and thermal decomposition of the material are modeled by the Pyropolis model described in detail in Refs. [2,11]. Both heat and mass transfer are assumed to proceed normally to the sample surface. To replicate the experimental conditions, we consider the two-layer structure including the layers of the combustible material and of the inert substrate with the thermal properties of plasterboard (k = 0.2 W/(m·K), ρ = 900 kg/m^3^, ρ = 950 J/(kg·K)). Conjugate heat transfer at the interface between the layers is accounted for via continuity of temperatures and heat fluxes. The interface, i.e., the rear surface of the combustible material layer, is set to be impermeable to the gas flow.

The model is incorporated into the ANSYS Fluent software, thereby enabling full coupling between the surface heat fluxes induced by the gaseous flame and the generation of volatiles fueling the flame. The pyrolysis model can also be used as a stand-alone model, i.e., separately from the CFD solver. In the latter case, surface heat fluxes at the specimen sides must be prescribed. Stand-alone simulations presented below were performed assuming a perfectly insulated back surface of the substrate while the incident radiative and convective fluxes at the exposed surface of the combustible material were set to 5 and 35 kW/m^2^, respectively. Although these boundary conditions do not exactly replicate the variety of the heating modes occurring in the experiment, use of these reference values makes it possible to evaluate the expected sensitivity of the simulation results to the model parameters.

Among the assumptions and simplifications made in the model formulation, we reiterate here that the heat transfer is only considered normally to the specimen surface, while the longitudinal transfer is ignored. Although this assumption works very well for the upward (concurrent) flame propagation mode, there is experimental evidence, e.g., Ref. [12], that longitudinal transfer may be more pronounced in a downwardly propagating flame (a detailed discussion can be found in Ref. [13]).

In the Pyropolis model, a combustible material undergoing thermal decomposition consists of virgin polymer, inert fiber (not present in the material studied here), and char (may not present in a non-charring material). Kinetic modeling of the material decomposition is described below in the next section. If a material is charring, then its intumescency or shrinking will greatly influence the effective thermal properties of the material. To quantify the degree of intumescency or shrinking of the material during its thermal degradation, the expansion factor, EF, is defined as the ratio of the final and initial specimen volumes. This quantity is considered as the material property and is assigned in the input data. For the material decomposing to produce char and gas volatiles, the final and initial volumes are the effective volumes of porous char and matrix (virgin) polymer. The expansion factor, therefore, is

(5) $$EF=\frac{V_{ch,eff}}{V_{mp,eff}}=\frac{V_{ch}/\left(1-\varphi_{ch}\right)}{V_{mp}/\left(1-\varphi_{mp}\right)}$$

where $V_{mp}$, $V_{ch}$ are the volumes occupied by the solid parts of the matrix polymer and char; and $\varphi_{mp}$, $\varphi_{ch}$ are the porosities of the matrix polymer and char. Using $V_{mp}=m_{mp}/\rho_{mp}$, $V_{ch}=m_{ch}/\rho_{ch}$ (where $m_{mp}$, $m_{ch}$ and $\rho_{mp}$, $\rho_{ch}$ are the masses and densities of the solid parts of the matrix polymer and char), and defining the mass fraction of gas volatiles as $\nu_{g}=\left(m_{mp}-m_{ch}\right)/m_{mp}$ (also the material property), we obtain the char porosity,

(6) $$\varphi_{ch}=1-\frac{1}{EF}\frac{\rho_{mp}\left(1-\varphi_{mp}\right)}{\rho_{ch}}\left(1-\nu_{g}\right)$$

corresponding to the given expansion factor. The char porosity strongly affects thermal properties (particularly the thermal conductivity) of the degrading material, which is evaluated as explained in Ref. [2]. Due to this, the simulation results appeared to be sensitive to the expansion factor pre-assumed in the simulations. Unless otherwise stated, we assume EF = 0.15 to allow for the significant shrinkage of the degrading material clearly observed in the experiment. To explore the effects incurred by these model parameters, the values of 0.075 and 0.3 have also been explored. Steady downward flame propagation was not predicted with the expansion factor above unity, i.e., in case if the material was assumed intumescent.

The experiment shows that the slab completely burns out, leaving a very small amount of solid residue behind the flame front (see Figure 1). At the same time, the MCC tests performed in nitrogen flow produced some amount of solid char (about 8% mass of the virgin material) after pyrolysis was complete. This implies that the char produced in pyrolysis is oxidized when the specimen burns in air. In the Pyropolis model, charring and non-charring materials are treated differently, but the char oxidation is not accounted for. In the simulations performed in this work, we considered material to be charring, and assumed the char yield corresponding to that recorded in the MCC tests. To approximately allow for the char oxidation, we used the heat of combustion of volatiles (20.8 MJ/kg), which is higher than the measured value of 19 MJ/kg and corresponds to the literature data for rigid polyurethane.

The effects of spatial resolution and the time step were carefully examined in the stand-alone simulations of the specimen heating and pyrolysis with the prescribed surface heat fluxes at the specimen sides. Uniform mesh with 60 cells across the specimen was found to ensure both the temperature gradient and the reaction zone in *the solid material* to be resolved accurately enough. In the substrate layer (plasterboard wall), the non-uniform mesh, 16 cells across the layer, was generated with the smallest cells adjacent to the rear specimen surface. The time step of 0.01 s was shown to provide the converged predictions (note that a smaller time step of 0.002 s was used in the gas phase simulations).

#### 3.2.2. Formal Kinetics of Material Decomposition

**Figure 9:**
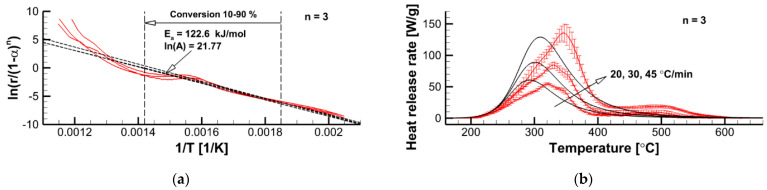
Determination of kinetic parameters in accordance with Equation (11) for the pre-assumed reaction order (**a**) and comparison of measured (solid lines with error bars) and simulated (solid lines, no error bars) dependencies $\dot{q}\left(T\right)$ at three heating rates (**b**).

The formal kinetic model does not consider the detailed chemistry of polymer pyrolysis but aims at replicating thermal decomposition of the material at microscale. To obtain the reference data, microscale combustion calorimetry (MCC) measurements were performed at three heating rates of 20, 30, and 45 K/min. The measurement results (averaged over several similar runs) are demonstrated in Figure 9b by solid lines with vertical bars showing the standard deviation of multiple tests. In this work, two formal kinetic models consisting of either the single global reaction or several independent reactions were derived and applied.

Potential applicability of the single-step global reaction model is guided by the observation that the measured signals (dependencies of heat release rate in volatile oxidation on sample temperature) do have a pronounced major peak. The single-step reaction model of the MCC test is given by the equality,

(7) $$\dot{q}\left(T\right)=\bar{\Delta q^{\prime }}\;\dot{r}\left(T\right),$$

where $\bar{\Delta q^{\prime }}$ = $\int_{0}^{\infty }\dot{q}\left(t\right)dt$ ≈ $\beta^{-1}\int_{T_{0}}^{T_{\max}}\dot{q}\left(T\right)dT$ is the total heat of volatile combustion per unit mass of the virgin material;

(8) $$\dot{r}\left(T\right)=Af\left(\alpha \right)\exp\left(-\frac{E_{a}}{ℛT}\right)$$

is the decomposition reaction rate; $\alpha =\int_{0}^{t}\dot{q}dt/\bar{\Delta q^{\prime }}$ is the heat-release-based conversion (0 ≤ α ≤ 1); $E_{a}$ and A are the apparent activation energy and corresponding pre-exponential factor; and f(α) is the conversion function allowing for the effect of conversion on decomposition reaction rate.

To simulate the dependence given by Equation (7), the following differential equations are solved numerically:

(9) $$\frac{dT}{dt}=\beta ,\;T\left(0\right)=T_{0},$$

(10) $$\dot{q}=\bar{\Delta q^{\prime }}\frac{d\alpha }{dt}=\bar{\Delta q^{\prime }}Af\left(\alpha \right)\exp\left(-\frac{E_{a}}{ℛT}\right),\;\alpha \left(0\right)=0.$$

where β is the sample heating rate. Formulation of the single-step global reaction model implies determination of $E_{a}$, A, and f(α). For the material investigated in this work, a conventional n-th order reaction model, $f\left(\alpha \right)={\left(1-\alpha \right)}^{n}$, was found to perform reasonably well (recall that the decomposition of many common polymers exhibits autocatalytic behavior and cannot be adequately replicated by the n-th order reaction model, see Ref. [14] for details). Assuming particular values of the rection order, n, and using the experimental dependencies of the decomposition reaction rate on temperature, $\dot{r}\left(T\right)=\dot{q}\left(T\right)/\bar{\Delta q^{\prime }}$, we plot the dependencies of $y=\dot{r}/{\left(1-\alpha \right)}^{n}$ on x=1/T, which are shown by the solid curves in the left plots in Figure 9 (each curve corresponds to one heating rate). These dependencies were then approximated by the straight lines,

(11) $$\ln\frac{\dot{r}}{{\left(1-\alpha \right)}^{n}}=-\frac{E_{a}}{ℛT}+\ln\left(A\right),$$

and the values of $E_{a}/ℛ$ by ln(A) were evaluated by the least squares method.

Each value of the pre-assumed reaction order relates to a particular pair of apparent activation energy and pre-exponential factor. Thus, the best triplet should be selected based on a comparison of the simulated dependence (7) (generated by solving Equations (9) and (10)) with the corresponding experimental curve.

In Figure 9a, the dependencies given by Equation (11) and those derived from the MCC measurement data are demonstrated for n = 3. Corresponding values of $E_{a}$ and ln(A) as well as a conversion range from 0.1 to 0.9 are also shown in Figure 9a. The plots in Figure 9b demonstrate a comparison of the measured dependencies $\dot{q}\left(T\right)$ with its simulated counterparts. This comparison indicates that the n-th order reaction model fails in the replication of all the patterns of the measured curves. While applying this approach for different pre-assumed reaction orders, we arrived at the following conclusions:

1. The first-order reaction model should be discarded for this experimental dataset.
2. For n ≈ 2 ($E_{a}$ = 76.8 kJ/mol), the simulated curves attain its maximum at approximately the same temperatures as the experimental counterparts.
3. Maximum reaction rates are best reproduced at n = 2.5 ($E_{a}$ = 98.9 kJ/mol).
4. The decomposition onset is best replicated at n = 3 ($E_{a}$ = 122.6 kJ/mol).
5. A further increase of the reaction order (n > 3) does not facilitate better agreement between the simulated and measured cures.

It is reasonable to expect that the velocity of the flame propagation should be controlled by the very onset of material decomposition and volatile emission. As such, for the pyrolysis reaction rate, $\dot{r}$ = $A{\left(1-\alpha \right)}^{n}\exp\left(-E_{a}/ℛT\right)$, we use n = 3, $E_{a}$ = 122.6 kJ/mol, and A = 2.84 × 10^9^ s^−1^. Although this model does not match the shape of the measured curves, it does properly replicate the pyrolysis onset, peak reaction rates, and the pyrolysis temperature intervals observed in the MCC tests at different heating rates.

A more accurate replication of the shape of the measured curves can be achieved by considering multiple decomposition reactions instead of a single one. This model still remains formal, since it does not consider the detailed chemistry of polymer decomposition but only aims at replication of the microscale measurements at different heating rates.

We formally represented the material of interest by a homogeneous additive blend of the several components (recall, this representation is not related to the actual composition of the material). In these components, the single-step reactions proceed independently.

We further assume that the heat of combustion of volatiles generated in all reactions is the same and is equal to that of the sample material, $\bar{\Delta q^{\prime }}$ = 19 kJ/g. Therefore, the heat release rate measured in the MCC tests is

(12) $$\dot{q}=\bar{\Delta q^{\prime }}\;\sum_{i}Y_{i,0}\;{\dot{r}}_{i},$$

where $Y_{i,0}$ is the initial mass fraction of the *i*-th component in the sample, and ${\dot{r}}_{i}$ is the *i*-th reaction rate.

The model of the MCC test consists of the equations similar to Equations (9) and (10), with α and $E_{a}$ replaced by $\alpha_{i}$ and $E_{a,i}$. To formulate the multi-reaction kinetic model, we have to determine $Y_{i,0}$, $A_{i}$, $f_{i}\left(\alpha_{i}\right)$, and $E_{a,i}$ for each reaction. After the measured dependencies $\dot{q}\left(T\right)$ are decomposed by the sum of several contributing curves formally corresponding to the individual reaction, each individual reaction was treated similarly to the single-step global reaction considered above.

**Figure 10:**
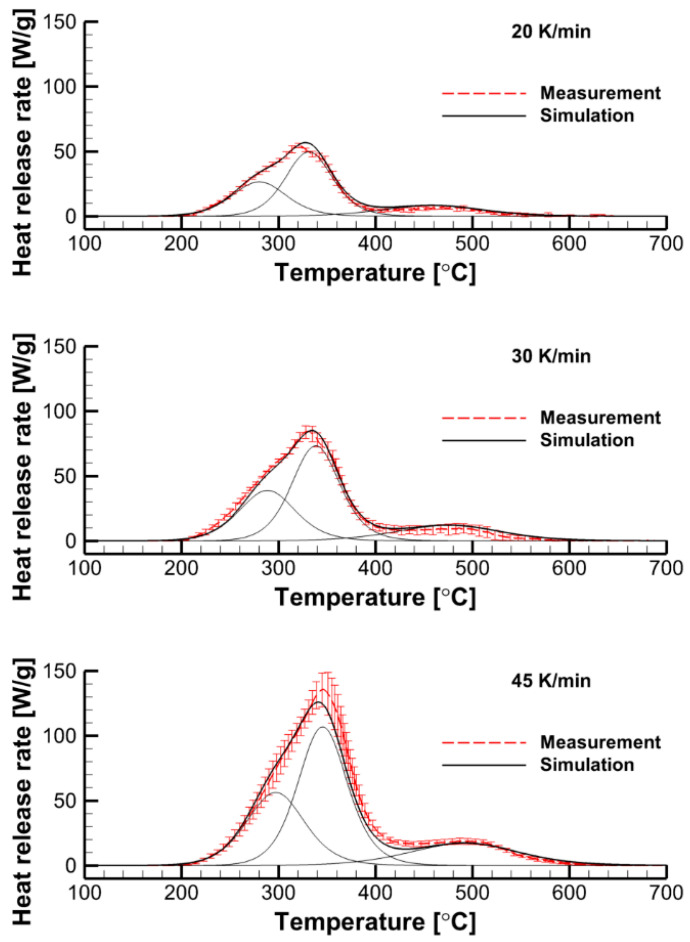
Performance of the three-reaction decomposition model: the simulations are compared with the MCC measurements at three heating rates of 20, 30, and 45 K/min. Thin lines show individual contributions by each reaction.

The simulated dependence, ${{\dot{q}}_{i}\left(T\right)|}_{Model}$, is obtained by solving ODEs (9) and (10) with the given kinetic parameters. In the case of interest, the best agreement was obtained for the kinetic parameters summarized in Table 1. As demonstrated in Figure 10, this kinetic model, which is formally composed by three independent reactions, replicates MCC measurement data at different heating rates at a very good accuracy, well exceeding that by the single-step global reaction model (see Figure 9).

**Figure 11:**
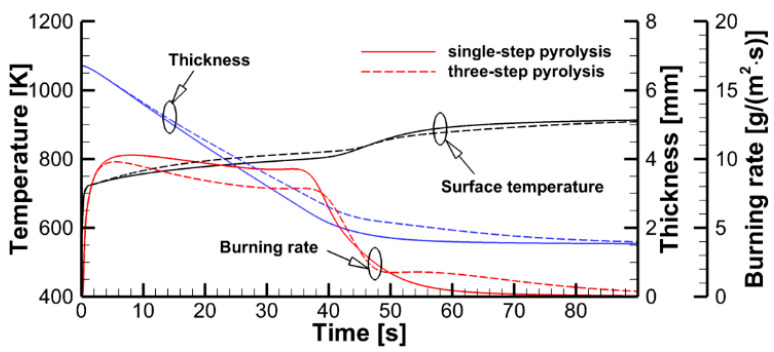
Stand-alone simulations of sample pyrolysis with the single-step global and the three-reaction models.

The effect of the pyrolysis model on sample decomposition is examined by performing the simulations using the pyrolysis model and prescribed heat fluxes at the sample surfaces. This comparison is presented in Figure 11, in which transient dependencies of predicted surface temperatures, current sample thicknesses, and volatile production rates are shown as simulated by the single-step global reaction model and by the three-reaction model described above. Although the selection of the kinetic model does affect the predictions, the overall effect appears to be moderate, and this effect weakens at a higher incident heat flux at the exposed sample surface. A weak sensitivity of the predictions to the accuracy of the kinetic model of the material decomposition highlights the governing role of the heat transfer from the exposed sample surface to the reaction zone. This conclusion, however, may fail near extinction, when the external heating is low, and the material decomposition kinetics becomes as important as the heat transfer to the reaction zone (see Ref. [15] for a simulation example).

### 3.3. Gas Phase Model

The laminar flow and flame are considered. The gas phase model (ANSYS Fluent 2021r2) includes the Navier–Stokes equations for the multicomponent reacting gas mixture, the ideal gas state equation, the single-step irreversible global reaction model for the oxidation of volatiles, the models for soot formation and oxidation, and the model to evaluate emission, transport, and absorption of thermal radiation. The details of the gas phase model can be found in Refs. [2,16].

The single-step irreversible global reaction of complete volatile oxidation is considered, and the volatiles are represented by the molecule CH_1.15_O_0.215_N_0.09_, corresponding to the atomic composition of the material. The reaction is assumed to be of the first order with respect to both fuel and oxygen (second order in total), with the activation energy 125.6 kJ/mol (30 kcal/mol) and the pre-exponential factor varied as explained in Section 4.1. Temperature- and concentration-dependent thermal properties of individual species and the mixture as well as the transport coefficients were evaluated.

For soot formation, the Moss–Brooks model [17] was applied as described in Ref. [18]. Soot oxidation was simulated using the Lee et al. [19] model (oxidation by molecular oxygen). Soot radiation is accounted for via the soot absorption coefficient proportional to the soot mass fraction and linearly dependent on temperature. The effective absorption coefficient by gaseous combustion products (CO_2_ and H_2_O) was evaluated using the weighted-sum-of-gray-gases model. The radiative transfer equation for the spectrum-averaged radiation intensities was solved by the finite-volume version of the discrete ordinates model. The flame radiative fraction is not prescribed, and radiative emission and absorption are directly resolved based on local temperature and composition.

## 4. Result

### 4.1. The Effect of Volatile Oxidation Kinetics

The simplified theory of flame spread (see Refs. [1,3,11] among others) is based on the assumption of an infinitely fast volatile oxidation reaction, which implies that the flame propagation velocity is only limited by the rate of heating the combustible material ahead of the flame front. Corresponding analytical relations for the steady flame propagation velocity in thermally thin and thermally thick limits are as follows:

(13) $$V_{f}=\{\begin{array}{c} \frac{k}{c_{s}\rho_{s}}\frac{T_{f}-T_{ign}}{T_{ign}-T_{0}}\frac{1}{\delta },\delta <\delta_{T}\\ \frac{4}{\pi }\frac{kc_{P}\rho }{k_{s}c_{s}\rho_{s}}{\left(\frac{T_{f}-T_{ign}}{T_{ign}-T_{0}}\right)}^{2}V_{g},\delta \geq \delta_{T} \end{array},$$

where k, $c_{P}$, and ρ are the gas thermal conductivity, specific heat, and density; $k_{s}$, $c_{s}$, and $\rho_{s}$ are the thermal properties of solid material; $V_{g}$ is the gas flow velocity; $T_{f}$ is the flame temperature; $T_{0}$ and $T_{ign}$ are the initial specimen temperature and the temperature of ignition; δ is the actual layer thickness; and

(14) $$\delta_{T}=\frac{\pi }{4}\frac{k_{s}}{c_{P}\rho V_{g}}\frac{T_{ign}-T_{0}}{T_{f}-T_{ign}}$$

is the characteristic thickness of the heated sub-layer.

This limit will hereafter be called the *thermal mode* of flame spread (recall that the heat transfer in burning solid material is also a controlling factor compared with the pyrolysis reaction rate). In this work, we performed simulations with the finite rate of volatile oxidation and, dissimilarly to the approximate theory, found the flame propagation velocity to be quite sensitive to the reaction rate in the gas phase. Such a sensitivity is an attribute of the *kinetic mode* of flame spread.

**Figure 12:**
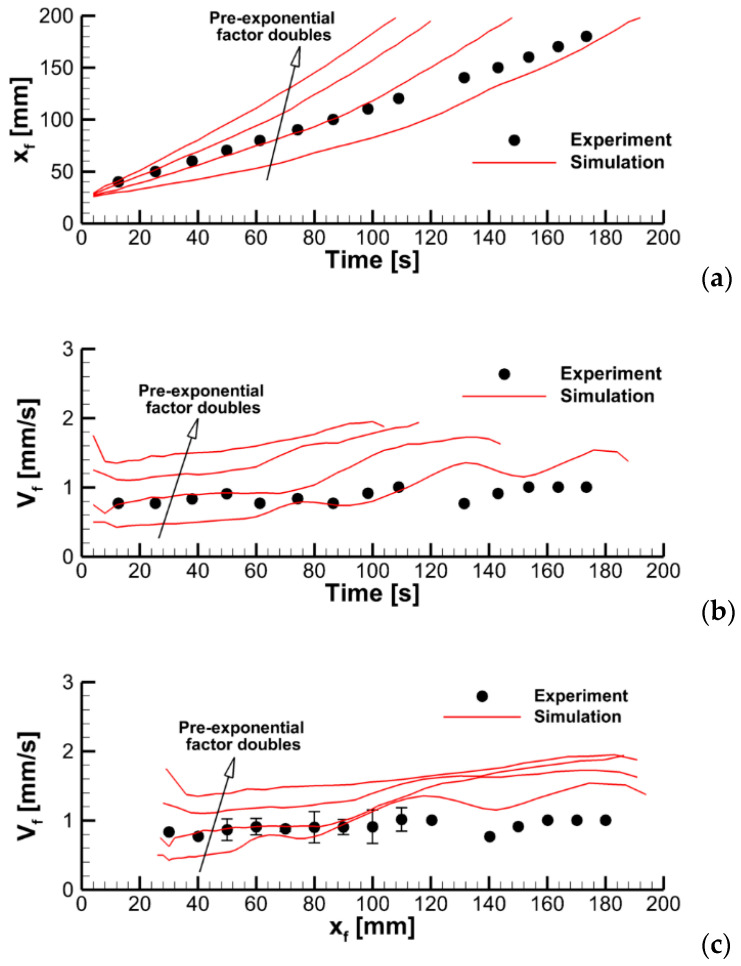
Dynamics of flame propagation in the axial plane: (**a**)—the pyrolysis front coordinate vs. time; (**b**)—front propagation velocity vs. time; (**c**)—flame propagation velocity vs. pyrolysis front coordinate. Solid lines—simulations with the pre-exponential factors of volatile oxidation reaction rate equal to 0.28 × 10^14^, 0.56 × 10^14^, 1.125 × 10^14^, and 2.25 × 10^14^ (m, mol, s).

To explore this effect, we varied the pre-exponential factor in the volatile oxidation reaction rate while keeping all the other model parameters unchanged. Predicted velocities of flame propagation are compared in Figure 12.

**Figure 13:**
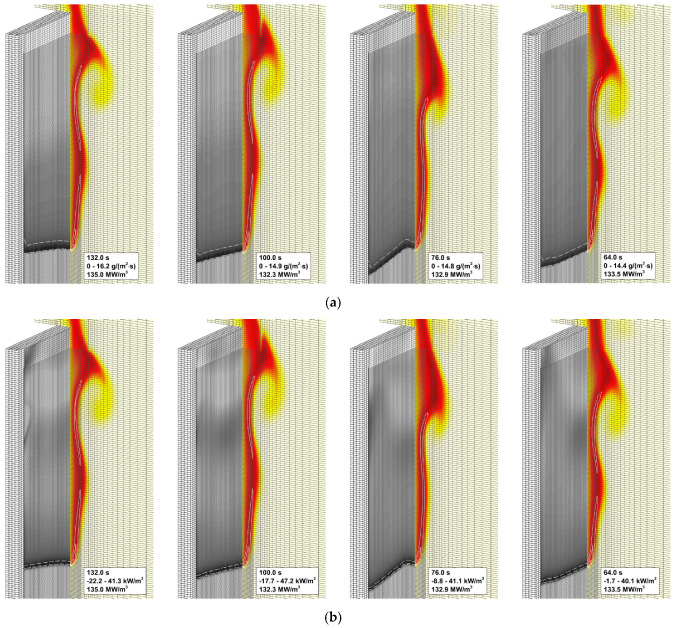
Instantaneous flame structure predicted with the different pre-exponential factors of volatile oxidation reaction rate. Left to right: the pre-exponential factor doubles. Solid lines: volumetric heat release rate in gaseous flame 20, 40, 60, 80, 100 MW/m^3^. (**a**) Burning rate at the sample surface. Dashed lines: mass loss rate 10 g/(m^2^·s). (**b**) Net heat flux at the sample surface. Dashed lines: net heat flux 35 kW/m^2^.

To explain the trend revealed in the simulations, the near-surface flame structure and distributions of the surface heat fluxes and the volatile production rate were examined first. In contrast with the expectations, neither predicted heat release rates in the gaseous flame distributions nor the heat fluxes and volatile emission at the sample surface appeared to observably increase with the increasing reaction rate in the flame. This observation is exemplified by Figure 13, which demonstrates the instantaneous distributions of the net heat flux and burning rate over the sample surface jointly with temperature and volumetric heat release at the vertical plane normal to the surface (time instants correspond to approximately the same location of the flame front). Despite these distributions being very similar, the predicted flame propagation velocities appear to be rather different as shown in Figure 12. This observation implies that the reason for the different velocities predicted with distinct pre-exponential factors of volatile oxidation reaction rate is not related to the material heating but is rather controlled by the burning mode in the gas phase; hence the reason for calling this mode of flame spread as the *kinetic* one.

A possible explanation is as follows. In the case of the finite-rate kinetics of volatile oxidation, a flammable gas mixture forms ahead of the flame, and the velocity of flame propagation becomes sensitive to the burning velocity in this mixture. Relative to the sample surface, the flame cannot propagate faster than the burning velocity less the velocity of the flame-induced oncoming upward buoyant airflow.

For the single-step global reaction of volatile oxidation, the laminar burning velocity in a homogeneous mixture is known to obey the following relation,

(15) $$S_{L}:\sqrt{D_{T}{\left(\frac{ℛT_{f}^{2}/E_{a}}{T_{f}-T_{0}}\right)}^{n}A\exp\left(-\frac{E_{a}}{ℛT_{f}}\right)},$$

where $D_{T}$ is the thermal diffusivity of the gas; $T_{f}$ is the flame temperature; and A and $E_{a}$ are the pre-exponential factor and apparent activation energy (n depends on the reaction orders, $n_{fuel}$ and $n_{O_{2}}$). Although the mixture is not homogeneous in the flame propagating over the combustible surface, Equation (15) can still be used for a qualitative analysis as an upper estimate if the stoichiometry is assumed. The finite value of $S_{L}$ implies that the flame propagation velocity relative to the sample surface is bounded by $S_{L}-V_{g}$, where $V_{g}$ is the velocity of the oncoming upward airflow near the burning surface. If $S_{L}\gg V_{g}$, then, in the kinetic mode of flame spread, the flame propagation velocity should scale as $A^{1/2}$.

**Figure 14:**
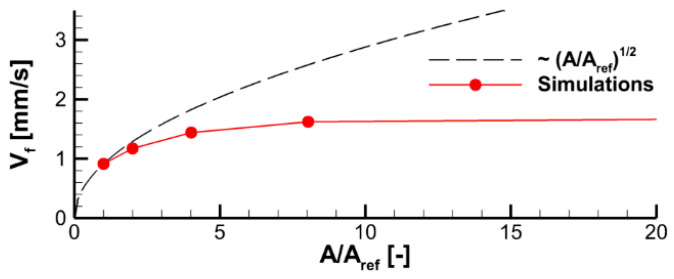
Effect of the pre-exponential factors of volatile oxidation reaction rate on velocity of flame propagation.

In Figure 14, the predicted flame propagation velocity is compared to the dependence proportional to $A^{1/2}$. It can be seen that the strongest dependence occurs at small values of $A^{1/2}$, i.e., in the kinetic limit. As the pre-exponential factor increases, a transition to the thermal mode occurs, and the flame propagation velocity ceases to depend on the pre-exponential factor, and, therefore, on the volatile oxidation reaction rate.

### 4.2. The Effect of Decomposition Kinetics of Combustible Material

To explore the effect of solid decomposition kinetics, we performed simulations with the single-step global reaction and the multistep-reaction model including three parallel independent reactions. For the single-step reaction, the kinetic parameters were n = 3, $E_{a}$ = 122.6 kJ/mol, and A = 2.84 × 10^9^ s^−1^ (the performance of this kinetic model is compared with the MCC measurement data at three heating rates in Figure 9). The kinetic parameters of the three-reaction model are provided in Table 1, and its ability to accurately replicate the MCC data is shown in Figure 10. A comparison of both kinetic models clearly shows the superiority of the three-reaction model in replicating the measurements *at microscale*.

**Figure 15:**
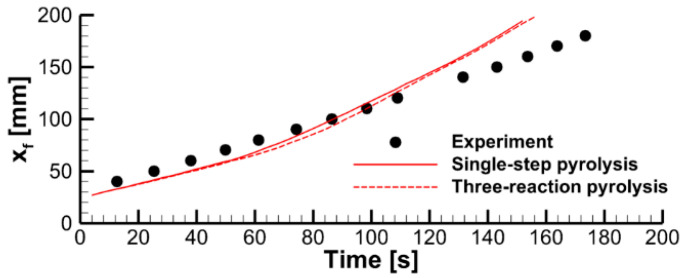
Pyrolysis front coordinate vs. time. Predictions obtained in coupled simulations with two kinetic models of solid pyrolysis are compared with the experimental data.

At the same time, the simulations of sample decomposition at a constant prescribed heat flux incident to the sample side surfaces (see Figure 11) revealed a relatively small difference in predicted decomposition dynamics. An even weaker effect of the material decomposition kinetic model on the predictions of flame spread can be deduced from Figure 15, demonstrating the dependence of the predicted and the measured flame front position on time.

Marginal sensitivity of the flame spread predictions to the kinetic model of solid pyrolysis indicates that the flame spread dynamics is controlled by the rate of heat transfer to the pyrolysis reaction zone rather than by the pyrolysis reaction rate. Therefore, trustable results can be obtained with the approximate single-step global reaction model.

**Figure 16:**
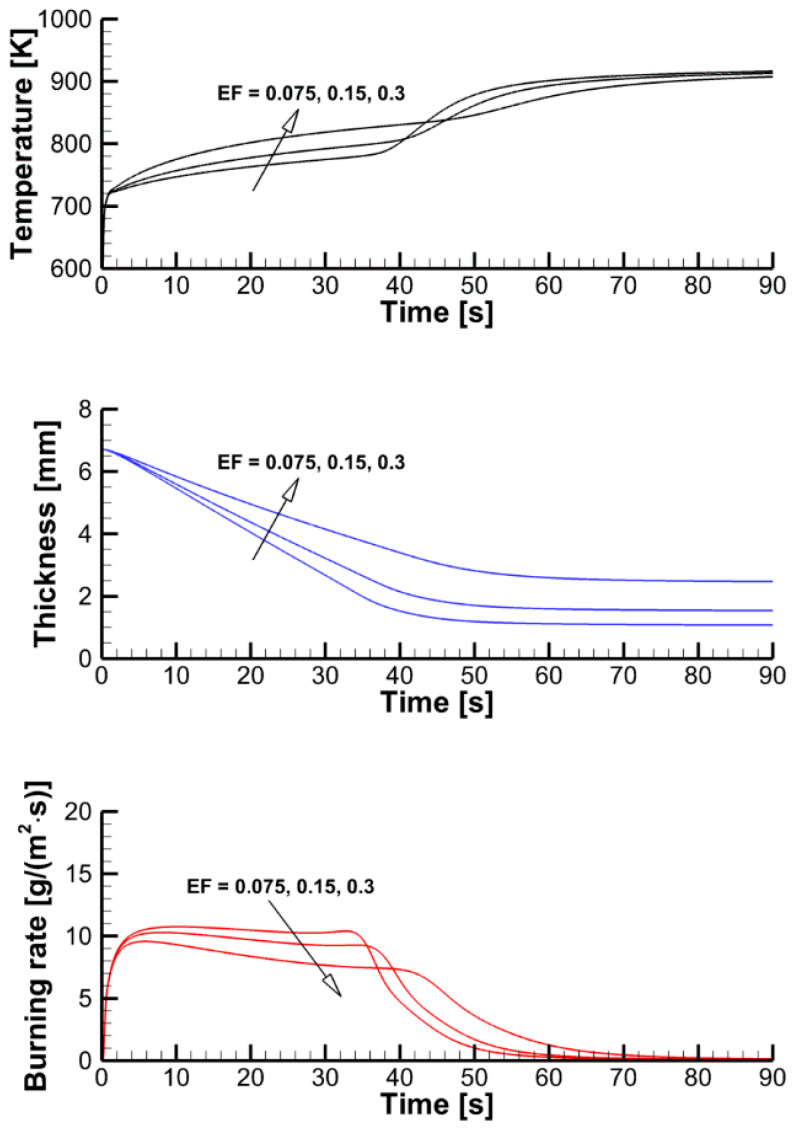
Transient variation of the temperature of the exposed surface, sample thickness, and mass loss rate predicted in simulations of sample heating and decomposition with prescribed surface incident heat fluxes and three values of the expansion factor.

The effect of the expansion factor (the model parameter defined by Equation (5) and prescribed in the input data) can be deduced from the stand-alone simulations performed with the constant surface heat fluxes and presented in Figure 16. A higher value of the expansion factor implies a thicker char layer separating the exposed sample surface and the pyrolysis reaction zone, thereby causing a slower burning-out of the sample at a lower burning rate.

**Figure 17:**
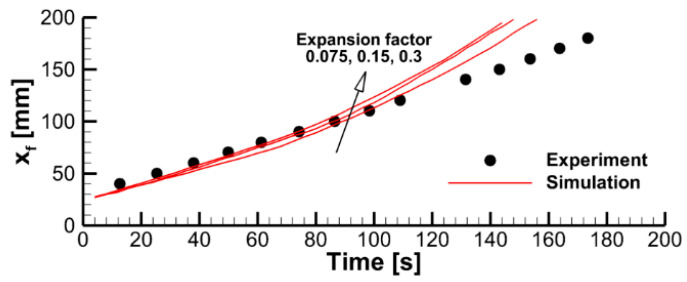
Pyrolysis front coordinate vs. time. Predictions obtained in coupled simulations with three values of the expansion factor are compared with the experimental data.

Coupled simulations of the flame spread and material decomposition revealed the same trend (see Figure 17), albeit not as pronounced as that in the simulations with constant prescribed surface heat fluxes.

Note that in this case, the variable called “expansion factor” is below unity and, therefore, it quantifies not expansion but shrinking of the material. When the expansion factor was set above unity (which is characteristic of intumescent materials), flame propagation was not observed in the simulations.

### 4.3. The Effect of External Heating

**Figure 18:**
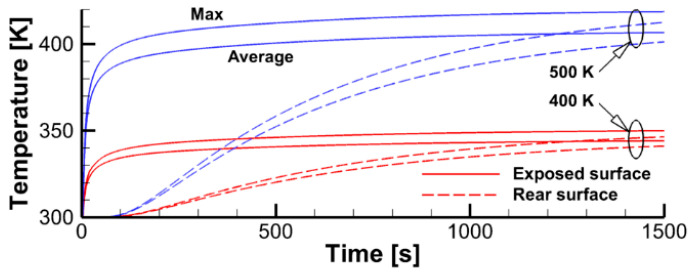
Transient variation of peak and surface-average temperatures for the sample impacted by thermal radiation from the heater. The heater temperature is indicated.

The simulations presented in this section were performed in two steps. In the first step, the specimen was exposed to the radiative flux produced by the heater, with no impact by the igniter. Transient variations of surface temperature at the exposed and rear sides of the sample shown in Figure 18 indicate that, in about 1500 s, the temperature distribution across the sample is approximately at the steady state.

**Figure 19:**
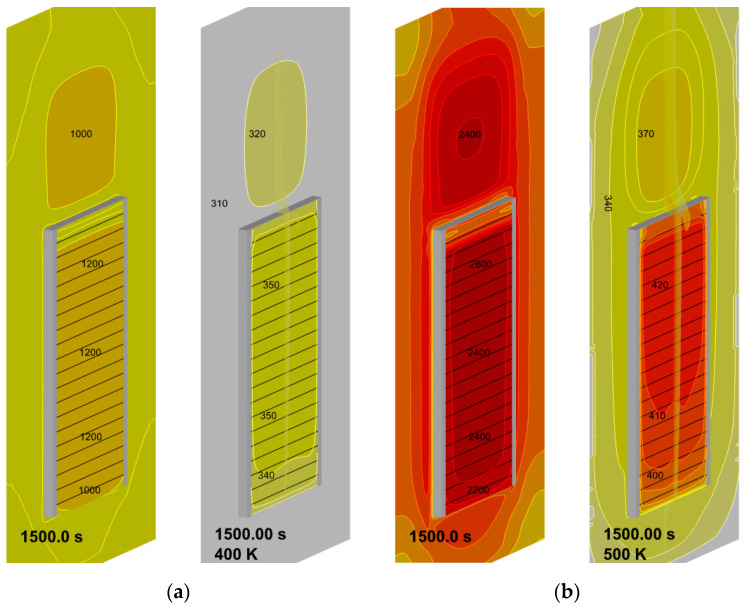
Steady-state surface distributions of incident radiation (**left**, the heat flux values are in W) and temperature (**right**, the temperature values are in K): (**a**)—heater temperature 400 K; (**b**)—heater temperature 500 K.

Simulated steady-state surface distributions of incident radiation and temperature presented in Figure 19 highlight the degree of their non-uniformity and show that the peak values of surface incident radiation are about 1.2 and 2.6 kW/m^2^ for the heater temperatures of 400 and 500 K (the corresponding surface temperatures are 350 and 420 K). Note that these values are, respectively, below and above the estimates derived above in Section 2.2.

**Figure 20:**
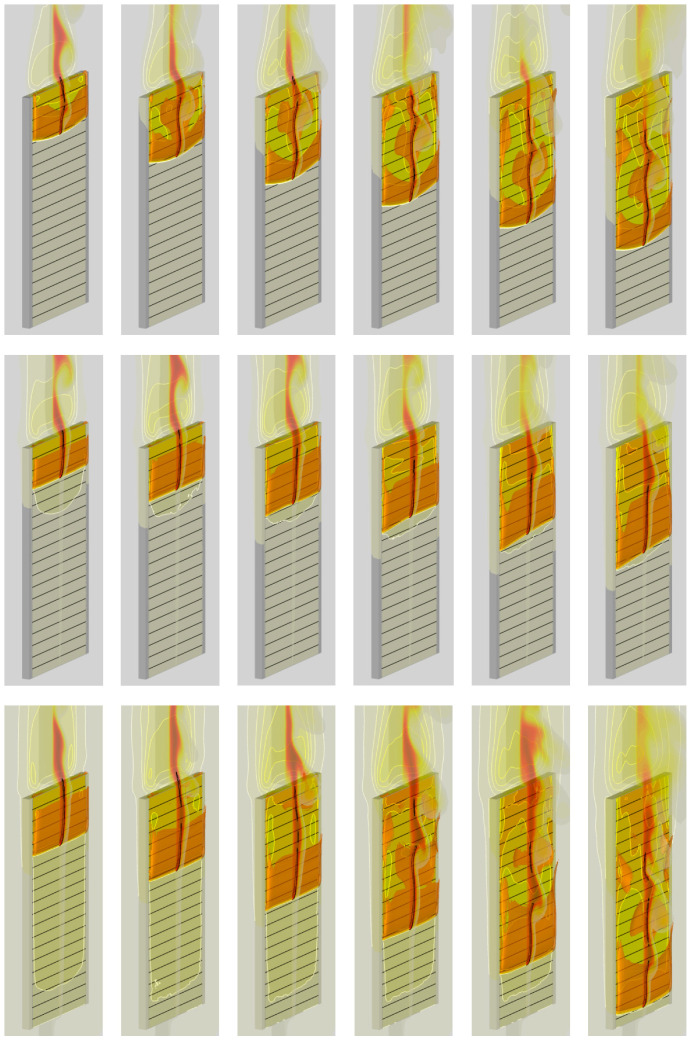
Predicted flame shape and instantaneous distributions of sample surface temperature and gas temperature at the vertical symmetry plane (with the heat release rate levels of 20, 40, 60, 80, 120 MW/m^3^) at time instants of 20, 40, 60, 80, 100, and 120 s. Heater temperature: **top**—300 K; **middle**—400 K; **bottom**—500 K.

**Figure 21:**
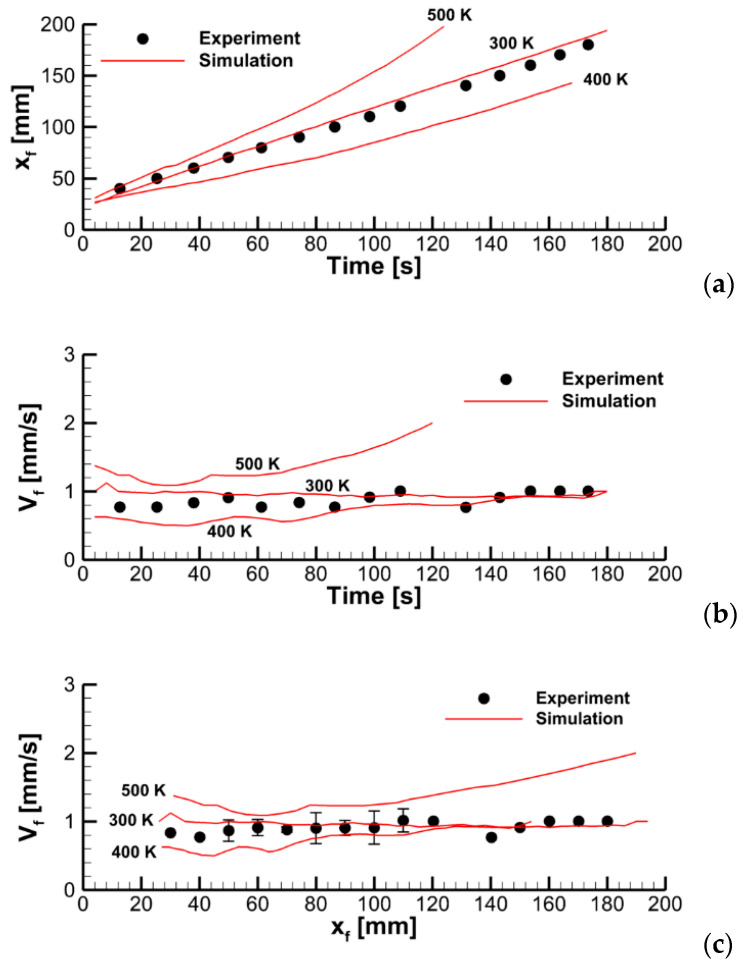
Dynamics of flame propagation in the axial plane: (**a**)—the pyrolysis front coordinate vs. time; (**b**)—front propagation velocity vs. time; (**c**)—flame propagation velocity vs. pyrolysis front coordinate. Solid lines—simulations with the different values of heater temperature.

In the second step, the igniter was activated, and sample ignition with subsequent flame spread was simulated for three heater temperatures of 300 (which coincides with the initial sample temperature and corresponds to the conditions of no external heating), 400, and 500 K. Simulation results, including the instantaneous flame shapes shown in Figure 20 and transient variations of the flame front coordinate and of the flame propagation velocity presented in Figure 21, demonstrate rather unexpected flame behavior for the intermediate case of the heater temperature of 400 K: the flame propagates slower than that with the heater temperature of 300 K.

**Figure 22:**
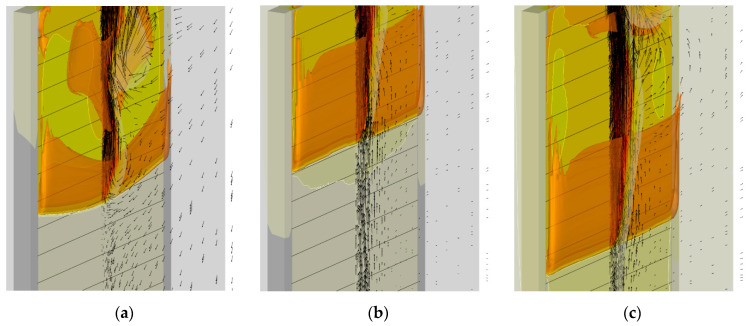
Predicted flow field near the flame front at time instant of 60 s. The same distributions are shown as those in Figure 20 jointly with the gas velocity vectors in the vertical symmetry plane. Heater temperature: (**a**)—300 K; (**b**)—400 K; (**c**)—500 K.

The explanation for this anomaly stems from the analysis of the flow field in and around the flame front. The development of the flow field with no external heating is qualitatively different when compared with that with heating. This difference is explained by the fact that without external heating, no buoyant flow is generated by the exposed surface far ahead of (i.e., below) the flame front, since the temperature gradient is absent. Yet an intensive buoyant flow is induced by the flame behind (i.e., above) the flame front. As a result, the zone ahead of the flame front is the area of impinging flow, which also generates the flow directed *downwards* (see Figure 22a). Such a downward flow facilitates flame propagation over the combustible surface. As soon as the external heating is turned on, the flow inversion occurs and the upward buoyant flow develops near the sample surface, which is clearly seen in Figure 22a,b.

It appears that this upward buoyant flow impedes the downward flame propagation whereas, in contrast, the sample temperature facilitates it. Thus, if the sample temperature is not sufficiently high (the case with a heater temperature 400 K), then the flame propagation velocity could be even lower than that with no external heating due to the formation of the uprising buoyant flow. This conclusion is only valid in case of the kinetic mode of flame spread. Note that, in contrast, according to the approximate theory, (see Equation (13)), the thermal mode implies that the flame propagation velocity will increase proportionally to the velocity of the flow incoming opposite to the direction of flame propagation.

### 4.4. Flame Impact on the Burning Material

**Figure 23:**
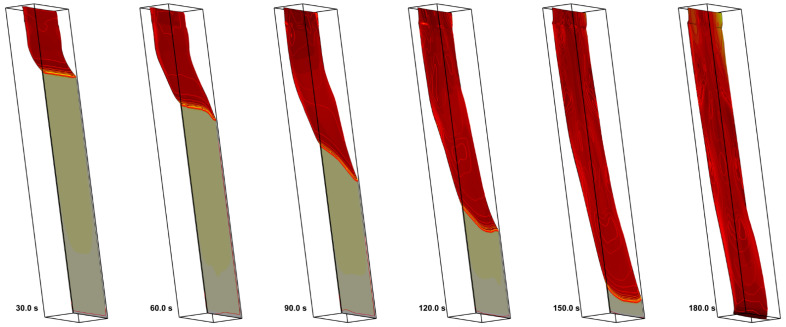
Visualization of downward flame spread at time instants in multiples of 30 s by variation of the layer thickness. The specimen surface is colored by the net heat flux received by the layer.

Propagation of the pyrolysis front over the specimen surface is visualized in Figure 23, where predicted specimen shapes with surface distributions of the temperature (color) and net heat flux (iso-lines) are presented at the time instants multiple of 30 s. The predicted length of the pyrolysis zone (the distance between the pyrolysis front and the point at which the virgin polymer is exhausted) correlates with the experimental observations, being similar to that shown in Figure 1 (right).

**Figure 24:**
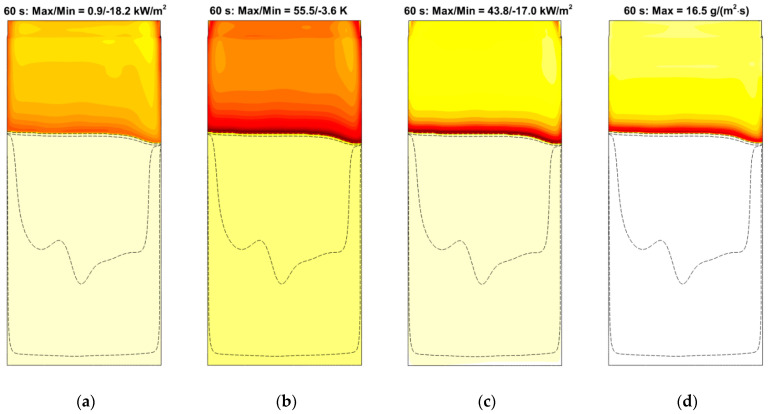
Distributions of radiative (**a**), convective (**b**), and net (**c**) heat fluxes and mass burning rate (**d**) over the specimen surface at time instant of 60 s. Dashed lines correspond to surface temperatures of 360, 380, 400, 600, 800, and 850 K. Maximum and minimum values of the heat fluxes and the burning rate are shown.

**Figure 25:**
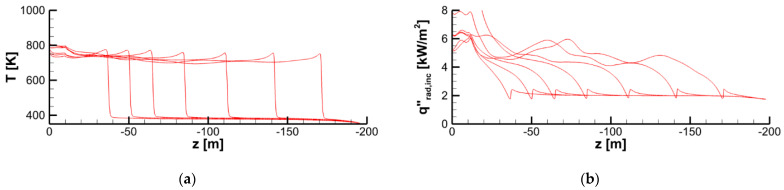
Distributions of temperature (**a**) and incident radiation (**b**) at the specimen surface along the vertical centerline at time instants of 20, 40, 60, 80, 100, 120, and 140 s.

**Figure 26:**
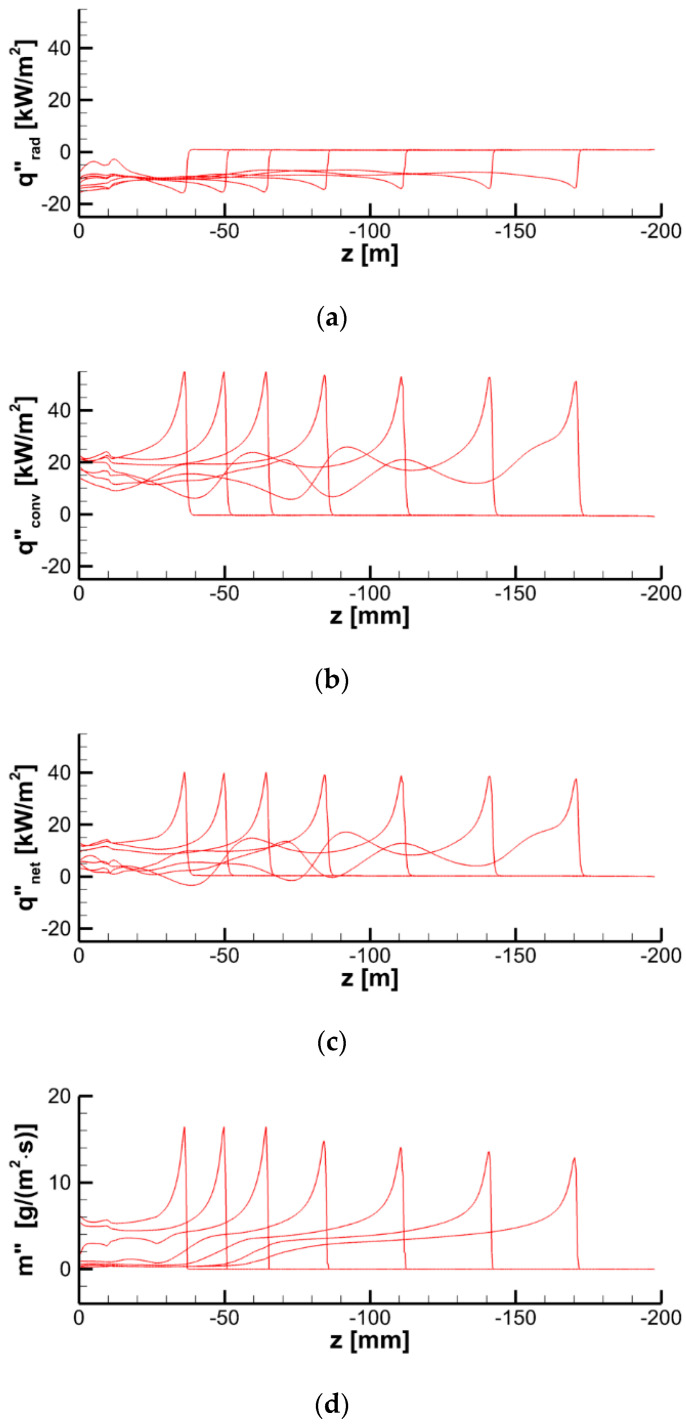
Distributions of radiative (**a**), convective (**b**), and net (**c**) heat fluxes and mass burning rate (**d**) at the specimen surface along the vertical centerline at time instants of 20, 40, 60, 80, 100, 120, and 140 s.

Spatial distributions of radiative, convective, and net heat fluxes, as well as of the mass burning rate over the specimen surface at a particular time instant are demonstrated in Figure 24, which shows the formation of the sharp pyrolysis front that separates the virgin material and the burning area. Distributions of the temperature, incident radiation, heat fluxes, and mass burning rate at the specimen surface along the vertical centerline at several consecutive time instants are provided in Figure 25 and Figure 26. All these quantities exhibit sharp peaks just behind the flame front. The spatial width of these peaks is determined by the thickness of the flame front in the gas phase. As the front passes ahead, these quantities relax to the plateau, which terminates as soon as the layer burns through.

The simulations have shown that predicted flame propagation velocity is very sensitive to and decreases with the pre-assumed specimen surface emissivity. This is the indication of the surface reradiation being of primary importance in the surface heat balance. Indeed, the radiative heat flux, ${q^{\prime \prime }}_{rad}=\varepsilon {q^{\prime \prime }}_{rad,inc}-\varepsilon \sigma T^{4}$, received by the part of the specimen that is engulfed in flame, is negative (see Figure 24a and Figure 26a). In its turn, it implies that the surface re-radiation, $\varepsilon \sigma T^{4}$, is greater than the absorbed radiative flux, $\varepsilon {q^{\prime \prime }}_{rad,inc}$.

Note that the incident radiation, ${q^{\prime \prime }}_{rad,inc}$, is provided by both the flame and the external heating by the radiative panel. As shown in Figure 25b, the radiative flux generated by the flame is somewhat higher than, albeit comparable to, that produced by the heater.

**Figure 27:**
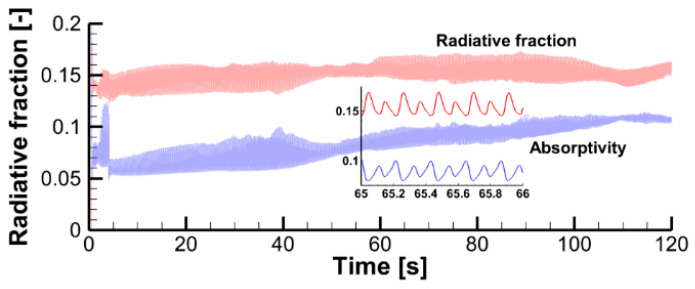
Predicted dependency of the radiative fraction and flame absorptivity.

In this scenario, the flame is optically thin and almost transparent for thermal radiation. This is supported by the observation that in the computational domain only 6 to 10% of the total radiative emission is absorbed by the flame (the total radiative fraction is predicted to be about 15%, see Figure 27). The radiative fraction and flame absorptivity are evaluated as:

(16) $$f_{r}=\frac{1}{\dot{Q}}\left(\int_{V}4\kappa \sigma T^{4}dV-\int_{V}\kappa GdV\right),$$

(17) $$a=\frac{\int_{V}\kappa GdV}{\int_{V}4\kappa \sigma T^{4}dV},$$

where κ is the effective absorption coefficient of combustion products (CO_2_, H_2_O, and soot), G is the incident radiation, and $\dot{Q}$ is the heat release rate.

As a result of flame transparency, the net heat flux, ${q^{\prime \prime }}_{net}={q^{\prime \prime }}_{rad}+{q^{\prime \prime }}_{conv}$, received by the layer in the preheat zone ahead of the flame edge mainly consists of the convective component. Convective and net surface heat fluxes as well as the mass burning rate attain their maximums in close vicinity of the flame edge (see Figure 24). The maximum values of convective and net heat fluxes are predicted to be 50–60 and 40–50 kW/m^2^, respectively. The maximum mass burning rate is below 20 g/(m^2^ s), and the maximum surface temperature is about 750–800 K as shown in Figure 25a.

## 5. Concluding Remarks

This study is among the very few three-dimensional simulations of downward flame spread, in which the gaseous flame is directly resolved and fully coupled with the volatile generation of solid fuel decomposition. The primary outcome of this work is in demonstrating ability of the model and three-dimensional computational framework (based on Pyropolys model coupled with ANSYS Fluent software) to replicate the experimentally observed dynamics of downward flame propagation over solid combustible material (rigid polyurethane). This is the important step in validating the model and establishing the requirements for the simulation methodology, since the downward flame propagation is a challenging task for computations.

A particular challenge for 3D computations is the need to provide a mesh resolution sufficient to resolve the temperature gradient and the inner structure of the reaction zone in the gaseous flame. The temperature gradient directly determines the heat flux from the flame edge to the preheat zone of the material, and the accurate resolution of the reaction zone is necessary to correctly predict the burning velocity of the volatiles-air mixture. The simulations revealed a strong sensitivity of the predictions to variation of thermal properties of the degrading material, which are controlled in this model via the expansion factor defined by Equation (5). For the self-sustained downward flame propagation to be predicted, the expansion factor must be set well below unity, which corresponds to a pronounced shrinking of the material upon heating and decomposition. It is this shrinking behavior that is indeed observed in the experiments. The specimen surface emissivity has been shown to strongly affect the predicted flame propagation velocity because of the considerable contribution of surface reradiation in the energy balance at the burning surface.

Two distinct modes of counterflow flame spread, thermal and kinetic, are identified. In the thermal mode, the flame propagation velocity is governed by the heating rate of the combustible material ahead of the flame front, whereas in the kinetic mode it is limited by the burning velocity of the gas mixture composed of pyrolysis volatiles and air ahead of the flame front. In the kinetic mode, the flame propagation velocity is sensitive to the reaction rate in the gaseous flame, whereas in the thermal mode, it is not.

An unexpected non-monotonic dependency of the flame propagation velocity on the external heating intensity has been observed in the simulations: if the specimen initial temperature incurred by the external radiation is not sufficiently high, then the flame propagation velocity could be even lower than that with no external heating. This counter-intuitive flame behavior is attributed to the flow inversion induced by the uprising buoyant flow, which overturns the downward portion of the flow that develops near the cold specimen surface.

In the simulations performed in this work, the kinetics of volatile oxidation in the gaseous flame has been pre-assumed rather than derived from independent measurements. At the same time, a good quantitative agreement has been achieved between numerically predicted and experimentally observed flame propagation dynamics, provided the specimen surface emissivity, material expansion factor, and volatile oxidation rate are jointly adjusted.

## Figures and Tables

**Table 1:** Kinetic parameters of three reactions used to approximate the MCC data.

Reaction	$n_{i}$	$\ln\left(A_{i}\right)$	$E_{a,i}$, kJ/mol	$Y_{i,0}$	$\bar{\Delta {q^{\prime }}_{i}}=Y_{i,0}\bar{\Delta q^{\prime }}$, kJ/kg
1	1.7	20.98	116.0	0.314	5.95
2	1.8	29.43	168.2	0.509	9.64
3	1.5	11.78	101.8	0.177	3.36

## Data Availability

Not applicable.

## Nomenclature

A	pre-exponential factor (s^−1^)
c	specific heat (J/(kg·K))
$E_{a}$	apparent activation energy (J/mol)
EF	expansion factor (-)
f(α)	conversion function (-)
G	incident radiation (kW/m^2^)
h	heat transfer coefficient (W/(m^2^·K))
k	thermal conductivity (W/(m·K))
$m^{\prime \prime }$	mass flux of volatiles at the specimen surface (kg/(m^2^·s))
M	molar mass (kg/mol)
$q^{\prime \prime }$	heat flux (W/m^2^)
$\dot{q}$	specific heat release rate per unit mass of virgin material (W/kg)
$\bar{\Delta q^{\prime }}$	heat of combustion of volatile oxidation per unit mass of virgin material (J/kg)
$\dot{r}$	pyrolysis reaction rate (s^−1^)
$\dot{R}$	matrix polymer decomposition rate ((kg/s)/m^2^)
ℛ	universal gas constant, 8.314 J/(mol∙K)
$S_{L}$	laminar burning velocity (m/s)
t	time (s)
V	volume of the mesh element (m^3^/m^2^)
$V_{f}$	flame propagation velocity (m/s)
$V_{g}$	gas flow velocity (m/s)
WF	weighting factor (-)
$x_{f}$	flame front coordinate (m)
Y	mass fraction (-)
Greek	
α	conversion (-)
β	heating rate (K/s)
ε	emissivity (-)
φ	porosity (-)
κ	absorption coefficient (m^−1^)
$\nu_{g}$	mass fraction of matrix polymer converted to volatiles (-)
ρ	density (kg/m^3^)
σ	Stefan–Boltzmann constant, 5.67 × 10^−8^ W/(m^2^·K^4^)
τ	time scale (s)
Subscripts	
c	convective
ch	char
cond	conductive
cr	critical
f	flame
fib	fiber
hfs	heat flux sensor
g	gas; gasification
inc	incident
i	solid component (*mp*, *fib*, *ch*)
j	reaction number
mp	matrix polymer
net	net value (radiative plus convective)
p	pyrolysis
r	radiative
res	solid residue
s	solid
surf	surface
TC	thermocouple
0	initial; ambient
∞	final
Abbreviations	
CFD	computational fluid dynamics
MCC	microscale combustion calorimetry
TGA	thermogravimetric analysis
TC	thermocouple

## References

[1] Quintiere J.G. (2002). Surface Flame Spread. SFPE Handbook of Fire Protection Engineering.

[2] Snegirev A.Y., Kuznetsov E.A., Korobeinichev O.P., Shmakov A.G., Trubachev S.A. (2021). Ignition and burning of the composite sample impacted by the Bunsen burner flame: A fully coupled simulation. Fire Saf. J..

[3] Quintiere J.G., Harkleroad M.T., Harmathy T. (1985). New Concepts for Measuring Flame Spread Properties. Fire Safety: Science and Engineering.

[4] Bhattacharjee S., King M.D., Paolini C. (2004). Structure of downward spreading flames: A comparison of numerical simulation, experimental results and a simplified parabolic theory. Combust. Theory Model..

[5] Long Y., Wichman I.S. (2009). Theoretical and numerical analysis of a spreading opposed-flow diffusion flame. Proc. R. Soc. A.

[6] Korobeinichev O.P., Karpov A.I., Bolkisev A.A., Shaklein A.A., Gonchikzhapov M.B., Paletsky A.A., Tereshchenko A.G., Shmakov A.G., Gerasimov I.E., Kumar A. (2019). An experimental and numerical study of thermal and chemical structure of downward flame spread over PMMA surface in still air. Proc. Combust. Inst..

[7] Korobeinichev O., Glaznev R., Karpov A., Shaklein A., Shmakov A., Paletsky A., Trubachev S., Hu Y., Wang X., Hu W. (2020). An experimental study and numerical simulation of horizontal flame spread over polyoxymethylene in still air. Fire Saf. J..

[8] Joshi A.K., Kumar A., Raghavan V., Trubachev S.A., Shmakov A.G., Korobeinichev O.P., Kumar P.B. (2021). Numerical and experimental study of downward flame spread along multiple parallel fuel sheets. Fire Saf. J..

[9] Korobeinichev O.P., Trubachev S.A., Joshi A.K., Kumar A., Paletsky A.A., Tereshchenko A.G., Shmakov A.G., Glaznev R.K., Raghavan V., Mebel A.M. (2021). Experimental and numerical studies of downward flame spread over PMMA with and without addition of tri phenyl phosphate. Proc. Combust. Inst..

[10] Kumar C., Kumar A. (2012). On the role of radiation and dimensionality in predicting flow opposed flame spread over thin fuels, Combust. Theory Model..

[11] Snegirev A., Talalov V., Stepanov V., Harris J. (2013). A new model to predict pyrolysis, ignition and burning of flammable materials in fire tests. Fire Saf. J..

[12] Ito A., Kashiwagi T. (1988). Characterization of flame spread over PMMA using holographic interferometry sample orientation effects. Combust. Flame.

[13] Wichman I.S. (1992). Theory of opposed-flow flame spread. Progr. Energy Combust. Sci..

[14] Snegirev A.Y., Talalov V.A., Stepanov V.V., Korobeinichev O.P., Gerasimov I.E., Shmakov A.G. (2017). Autocatalysis in thermal decomposition of polymers. Polym. Degrad. Stability.

[15] Snegirev A., Talalov V., Stepanov V., Harris J. (2013). Formal kinetics of polymer pyrolysis for modeling of ignition and burning in fire tests. Proceedings of the 13th International Conference Interflam 2013, Royal Holloway College University ofLondon.

[16] Kuznetsov E.A., Snegirev A.Y., Markus E.S. (2020). Radiative extinction of laminar diffusion flame above the flat porous burner in microgravity: A computational study. Combust. Explos. Shock Waves.

[17] Brookes S.J., Moss J.B. (1999). Prediction of Soot and Thermal Radiation in Confined Turbulent Jet Diffusion Flames. Combust. Flame.

[18] Snegirev A., Markus E., Kuznetsov E., Harris J., Wu T. (2018). On soot and radiation modeling in buoyant turbulent diffusion flames. Heat and Mass Transfer.

[19] Lee K.B., Thring M.W., Beer J.M. (1962). On the Rate of Combustion of Soot in a Laminar Soot Flame, Combust. Flame.

